# Role of membrane biophysics in Alzheimer's–related cell pathways

**DOI:** 10.3389/fnins.2015.00186

**Published:** 2015-05-27

**Authors:** Donghui Zhu, Brittani L. Bungart, Xiaoguang Yang, Zhaxybay Zhumadilov, James C-M. Lee, Sholpan Askarova

**Affiliations:** ^1^Department of Chemical, Biological and Bioengineering, North Carolina A&T State UniversityGreensboro, NC, USA; ^2^Indiana University School of Medicine Medical Scientist Training Program, Indiana University School of MedicineIndianapolis, IN, USA; ^3^Department of Clinical Neuroscience and Rehabilitation, Institute of Neuroscience and Physiology, Sahlgrenska Academy at University of GothenburgGothenburg, Sweden; ^4^The Hope Center for Neurological Disorders and Department of Neurology, Washington University School of MedicineSt. Louis, MO, USA; ^5^Department of Bioengineering and Regenerative Medicine, Center for Life Sciences, Nazarbayev UniversityAstana, Kazakhstan; ^6^Department of Bioengineering, University of Illinois at ChicagoChicago, IL, USA

**Keywords:** amyloid-β peptide, amyloid precursor protein, membrane molecular order, membrane fluidity, cerebral endothelium

## Abstract

Cellular membrane alterations are commonly observed in many diseases, including Alzheimer's disease (AD). Membrane biophysical properties, such as membrane molecular order, membrane fluidity, organization of lipid rafts, and adhesion between membrane and cytoskeleton, play an important role in various cellular activities and functions. While membrane biophysics impacts a broad range of cellular pathways, this review addresses the role of membrane biophysics in amyloid-β peptide aggregation, Aβ-induced oxidative pathways, amyloid precursor protein processing, and cerebral endothelial functions in AD. Understanding the mechanism(s) underlying the effects of cell membrane properties on cellular processes should shed light on the development of new preventive and therapeutic strategies for this devastating disease.

## Introduction

Alzheimer's disease will claim 13.2 million Americans by 2050 if no preventive treatments are found. The increasing number of AD victims puts a heavy economic and emotional burden on society, and thus AD has become an urgent national health and research priority. AD is complicated and multi-factorial involving numerous etiopathogenic mechanisms. Therefore, it is unlikely that any one single intervention well be efficacious to delay, prevent, or cure it. Many mechanisms involved in the pathogenesis and pathophysiology of AD have yet to be elucidated. In fact, recent studies provide strong evidence that cell membrane composition and cell biophysics play an important role in a number of pathophysiological events in AD (Hicks et al., 2012).

Cellular membrane lipid composition is dynamically changing and correlated with the progression of AD (Frisardi et al., 2011). In addition, alterations of membrane cytoskeleton may change the mechanical properties of cells and cell membranes, leading to eventual changes of cell functions, such as adhesion. Although, recent research findings show that the influences of membrane lipids and properties have been proven in many cellular pathways and processing implicated in AD, the role of altered lipid composition and membrane properties in the disease has yet to be fully elucidated.

Another important area of research investigates which aggregated forms of amyloid-β peptide (Aβ) are involved in the pathogenesis of AD. In fact, recent studies provide evidence showing that Aβ oligomers trigger many downstream oxidative pathways and neuro-inflammation (Salminen et al., 2009; Hodgson et al., 2013; Meraz-Rios et al., 2014). In this review, we address Aβ-related cellular processes in their relation to the physical properties of the cell membranes. Specifically, we summarize the role of biophysical factors in Aβ aggregation and the effects of oxidative stress and Aβ on membrane biophysics, membrane biophysics on amyloid precursor protein (APP) processing, and membrane mechanics in altered endothelial functions and blood brain barrier (BBB).

## The biophysics of Aβ aggregation

**Figure 1:**
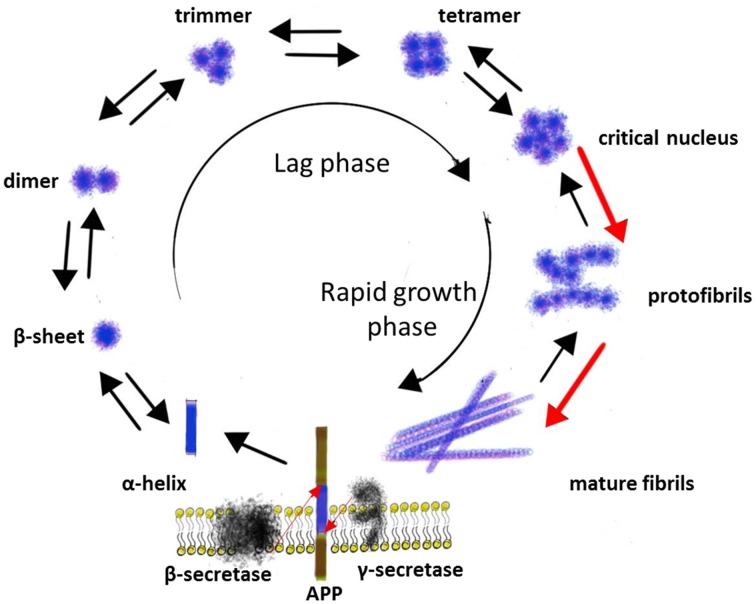
**The aggregation of Aβ**.

Amyloidogenic processing of the APP by β- and γ-secretase leads to the production of Aβ monomers of different lengths, of which the Aβ_1−40_ is the major species and the Aβ_1−42_ is the most fibrillogenic and predominant component in AD plaques (Bernstein et al., 2005). Aβ_1−40_ and Aβ_1−42_ consist of hydrophilic N-terminal regions (residues 1–28) and hydrophobic C-terminal regions (residues 29–40 or 29–42), which is originally the part of a transmembrane α-helix of APP (Figure 1). *In vitro* studies have demonstrated that Aβ monomers can exist in three major conformation forms: α-helix, β-sheet or random coil (Liu et al., 2006). Since the fibrils mostly consist of β-sheets, while the original hydrophobic part of Aβ is an α-helix, the conformational transition of Aβ from α-helix to β-sheet probably is the very first step in fibril formation, and there is evidence that conformational transitions of Aβ monomers depend on physical and chemical parameters of the environment. For example, in aqueous solution Aβ exists as a mixture of all three conformation forms; in fluorinated alcohols, as well as at pH 1-4 and 7-10, Aβ exists in a form of α-helix, while β-sheet favored at pH 4-7 (Liu et al., 2006). In turn, the hydrophobic and positively charged self-assembled lipid monolayers (SAM) induce more Aβ adsorption, faster β-sheet formation, and stronger binding affinity than the hydrophilic and the negatively charged SAM. All of them accelerate Aβ aggregation and promote the structural conversion from an unstructured conformation to a β-sheet-containing structures compared to neutral solutions (Wang et al., 2011).

The next step in a process of Aβ aggregation is forming hydrogen bonds between the amide and the carbonyl (check spell) groups of anti-parallel oriented β-sheets with further aggregation into higher order structures (Poduslo and Howell, 2015). The aggregation of Aβ into fibrils is a complicated multi-step process that occurs through a number of intermediate structural forms and can be described as a sequential process consisting of several phases: monomers → misfolded monomers → soluble oligomers (clusters of small numbers of peptide molecules without a fibrillar structure) → protofibrils (aggregates of isolated or clustered spherical beads made up of ~20 molecules with β-sheet structure) → mature fibrils (Sengupta et al., 2014) (Figure 1).

There is evidence that the formation of Aβ fibrils is a nucleation-depended process. The conversion of a peptide into a fibril includes a lag phase that reflects the thermodynamic barrier to the formation of a nucleus followed by a rapid growth phase involving the sequential incorporation of Aβ peptides, producing rigid structures consisting of several layers of cross-β sheets. Several studies indicated that monomer, dimer, trimer, and tetramer species of Aβ exist in a rapid equilibrium, while pentameric or hexameric aggregates form a critical nucleus for higher order assembly (Figure 1) (Mclaurin et al., 2000; Chiti and Dobson, 2006).

Several studies have indicated that the size and the shape of Aβ aggregates, as well as the kinetics of their formation, depend on the physicochemical nature of the surface. For example, on hydrophilic surface (mica) Aβ formed particulate, pseudomicellar aggregates, while on hydrophobic surface Aβ formed uniform, elongated sheets with the dimensions consistent of β-sheets (Kowalewski and Holtzman, 1999). These studies suggest that Aβ fibril formation may be driven by interactions at the interface of aqueous solutions and hydrophobic substrates, as occurs in membranes and lipoprotein particles *in vivo* (Kowalewski and Holtzman, 1999; Ban et al., 2006; Wang et al., 2011). In turn, metal ions have been shown to play a role in Aβ aggregation. Some studies have indicated that very low levels of copper and zinc initiate seeding and oligomerization of Aβ and accelerate fibril formation by unfolding the helical structure in Aβ peptide and stabilizing the formation of vital salt-bridges within and between Aβ peptides (Mclaurin et al., 2000; Pan and Patterson, 2013). Calcium has been also shown to accelerate both the formation of protofibrils and its conversion to fibrills (Isaacs et al., 2006). On the other hand, there is evidence that many membrane associated proteins and glycolipids can regulate Aβ conformation and aggregation in both enhancement and inhibition manner as well (Mclaurin et al., 2000). For example, the binding of Aβ to the membrane sialoglycolipid GM1 ganglioside can lead to a conformational change in Aβ and seeding of aggregation (Manna and Mukhopadhyay, 2013). Aβ interactions with phospholipids can also induce a structural transition from random coil to β-sheet in Aβ40/42 and Aβ25-35, increase the local peptide concentration (in a vesicular environment) and accelerate fibril formation (Yuyama et al., 2008).

Thus, aggregation of Aβ is a multistep process which can be modulated by physical and chemical factors such as conformation of Aβ, pH, electrical charge, hydrophilicity, or hydrophobicity of the environment, and interaction with other elements that either promote or inhibit Aβ aggregation (Mclaurin et al., 2000). Identification of these factors and understanding the driving forces behind these interactions will help to reveal new therapeutic targets for prevention of amyloid formation and its associated toxicity (Mckoy et al., 2012; Luo et al., 2014; Yang et al., 2014). Since aggregation of Aβ occurs mainly at the surface of the cell membranes in next chapter the influence of the cell membrane's composition and biophysics on Aβ aggregation will be discussed.

## Aggregation of Aβ peptide is influenced by the membrane's composition and biophysics

The cellular membrane is known to be a highly dynamic structure with different lateral phases, which are mostly dependent upon membrane composition, but also temperature and pH if a deviation from physiological condition is present (Heberle and Feigenson, 2011). One of these phases, commonly referred to as the lipid raft, has a high molecular order which is more tightly packed than non-raft domains due to intermolecular hydrogen bonding involving sphingolipids and cholesterol (Barenholz, 2004; Fantini and Yahi, 2010). They are also known to be the platform for the assembly of many signaling molecules.

The formation of these microdomains is attributed to the protein-protein and protein-lipid synergy, which induces the observed high molecular order (Pike, 2006). For example, cholesterol, found in substantial concentrations in lipid rafts, is known to decrease lateral lipid diffusion, while increasing the surface hydrophobicity (Yu and Zheng, 2012). In the bilayer membrane and vesicle models, lipid rafts are found to coexist with fluid lipid regions, which are composed mainly of homogenous phosphatidylcholine (PC) and cholesterol (Dietrich et al., 2001).

In the pathology of AD, lipid rafts are known to be the main location for the interaction of Aβ with the cellular membrane (Kim et al., 2006; Choucair et al., 2007; Williamson et al., 2008; Cecchi et al., 2009; Ogawa et al., 2011; Mazargui et al., 2012). Two of the components of lipid rafts, cholesterol and the ganglioside called monosialotetrahexosylganglioside (GM1), have been given most of the spotlight in this interplay. Due to shingolipids having saturated fatty acids as side chains and cholesterol being able to pack closely with them, lipid rafts are of a higher order and have less fluid in the hydrophobic region than the surrounding domains (Thakur et al., 2011). Cholesterol then, in turn, is important in regulating the formation and function of lipid rafts and in increasing the concentration of GM1 in these microdomains (Cecchi et al., 2009).

When the cholesterol concentration is manipulated, a noticeable difference in the cellular membrane's influence on Aβ occurs. The concentration of cholesterol in lipid rafts correlates to the adsorption of Aβ_1−42_ on a 1-palmitoyl,2-oleoyl-sn-glycero-3-phosphocholine bilayer, while having little affect in a dipalmitoylphosphatidylglycerol bilayer model (Thakur et al., 2011). Any change in the cholesterol of the cellular membrane in human neuroblastoma cells results in causing Aβ_1−42_ oligomers to react differently with the bilayer (Williamson et al., 2008). In fact, it has been reported that a higher cholesterol content in the bilayer leads to increased membrane rigidity, which is more favorable for Aβ-membrane interactions, and a concomitant increase in the accumulation of Aβ_1−42_ (Yip et al., 2002).

When Aβ adsorbs or inserts into the membrane bilayer, the peptide-lipid interactions make the secondary and tertiary structure change to a more energetically favorable configuration (Zhao et al., 2011). Aβ was also observed to accumulate in size from monomer to hexamer and beyond, with the majority of the aggregates' size in the dimer to tetramer range. In comparison of the size of aggregate created in and not in the presence of cellular membrane, the larger aggregates are formed when the cell membrane is involved (Johnson et al., 2011). In neutral solution, Aβ consists of mostly alpha-helix or random coil. When Aβ is incubated with lipid rafts acquired from rat neuronal cells, cholesterol affects the conversion of the α-helix conformation of Aβ to the β-sheet-rich structure, which is synonymous with toxicity (Zhao et al., 2011).

Molecular dynamics (MD) simulations uncovered another vindication for the confirmation change of Aβ_1−40_ and Aβ_1−42_. The presence of GM1 amplifies the conversion of the α-helix to principally β-sheets through the formation of beta-hairpins at the C-terminal (Ogawa et al., 2011). This dependence on GM1 was confirmed when 5 μM Aβ_1−42_ preferentially aggregates in vicinity to GM1-liposomes. On the other hand, 5 μM Aβ_1−40_ did not aggregate under these conditions, showing that Aβ_1−40_ and Aβ_1−42_ interact dissimilarly with cellular membrane (Ogawa et al., 2011). In fact, Aβ_1−42_ accumulates more than Aβ_1−40_ in mice brains (Abramowski et al., 2012; Manna and Mukhopadhyay, 2013). Furthermore, in lipid rafts with no GM1, only a portion versus all of the membrane-associated Aβ obtains the beta-sheet folds (Lemkul and Bevan, 2011).

GM1 has also undergone similar scrutiny as cholesterol in its capacity to induce Aβ binding to the cell membrane. Gangliosides, such as GM1, have the ability to host the location for Aβ to seed (Kakio et al., 2002; Mazargui et al., 2012) and are even required for oligomerization to occur, while lipid and cholesterol is not necessary (Kim et al., 2006). In mice neuronal cells, Aβ was found to colocalize with GM1 (Williamson et al., 2008; Hong et al., 2014), which in fact can be partially inhibited by cholesterol (Williamson et al., 2008). Aggregation of Aβ_1−40_ occurs during the co-incubation of Aβ_1−40_ with raft-like liposomes containing GM1, cholesterol, and sphingomyelin. Additionally, aggregation is not elicited during the incubation of non-raft-like structures, such as GM1 with PC, but is when raft-like structures, such as cholesterol with GM1 and PC, are involved, further proving that Aβ depends upon an environment such as the lipid raft to cumulate (Okada et al., 2008).

Other factors in relation to the direct composition of the cellular membrane are investigated to determine their influence on Aβ adsorption and intercalation, such as the cell membrane's surface charge. Anionic areas in human neuroblastoma cellular membrane promote the interactions of Aβ_1−40_(Johnson et al., 2011), while aggregation is inhibited in the presence of vesicles with negatively charged surfaces. Adsorption of Aβ_1−40_ is more likely to occur on a negatively charged surface, and with neutral membranes experience mostly insertion (Sabaté et al., 2012). This suggests that dynamic interactions mediate this, and it is not only dependent upon the negative charge of the membrane as shown in vesicle-model studies.

The cellular membrane's biophysics are central in the actions that Aβ undergoes, which can be explained by Aβ being an amphipathic molecule that will adsorb non-specifically at an air and water interface and penetrate into uncompressed monolayers formed by zwitterionic and anionic phospholipids (Relini et al., 2009). The influence of the biophysical parameters and cell membrane's composition on Aβ conformational state and aggregation is summarized in the Table 1. However, this is not a one-way interaction; Aβ also influences the cellular membrane's biophysics, which will be discussed in the following chapter.

**Table 1:** **The influence of the cell membrane's composition and biophysics on Aβ conformational state and aggregation**.

**Physical and chemical factors**	**Conformational state of Aβ**	**Aggregation of Aβ**	**References**
pH 1-4	β-sheet	↑	Liu et al., 2006; Bhowmik et al., 2014
pH 7-10	ά-helix	↓	Liu et al., 2006; Bhowmik et al., 2014
Positive charge	β-sheet	↑	Wang et al., 2011
Electrically neutral environment	ά-helix	↓	Wang et al., 2011
Hydrophobic environment	β-sheet	↑	Wang et al., 2011
Hydrophilic environment	ά-helix	↓	Kowalewski and Holtzman, 1999; Liu et al., 2006; Wang et al., 2011
Membrane fluidity ↓	NA	↑	Yip et al., 2002
Membrane fluidity ↑	NA	↓	Yip et al., 2002
Cholesterol	β-sheet	↑	Yip et al., 2002; Schneider et al., 2006; Zhao et al., 2011
GM1	β-sheet	↑	Kim et al., 2006; Ogawa et al., 2011; Manna and Mukhopadhyay, 2013
Phospholipids	β-sheet	↑	Yuyama et al., 2008
Metal ions	unfold ά-helix	↑	Mclaurin et al., 2000; Isaacs et al., 2006; Pan and Patterson, 2013; Yang et al., 2014

*↑ denotes increase, ↓ denotes decrease, NC denotes no change, NA denotes data not available*.

## The membrane's composition and biophysics is influenced by Aβ peptide and oxidative stress

All of the aspects, which evoke the aggregation of Aβ, of the cell membrane's biophysics mentioned in the previous section can also be impacted due to Aβ interacting with the cellular membrane. Aβ has been observed to alter the fluidity and molecular order of the cellular membrane, along with acting in a functional manner by having the ability to create a pore when insertion occurs. Other features are also affected that were not discussed, such as the conductance of the cell membrane and the orientation of the lipid tails and cholesterol.

When Aβ integrates into the cellular membrane, an invagination causes voids, which fill with excess water and protein, engendering the cell membrane to become more molecularly disordered (Chang et al., 2010). In one scenario, Aβ_1−42_ causes an increase in the amount of water in the hydrophobic region of the bilayer in neuronal cells (Hicks et al., 2008). This alteration of the molecular order has been proposed to be the mechanism to explain the decrease in thickness of the lipid bilayer in the companionship of Aβ. By using MD simulations of Aβ insertion into the membrane bilayer to observe the molecular effects, the electrostatic forces cause the most thermodynamically stable orientation for the lipids and cholesterol to be different. The adjacent cholesterol tilts, which is not observed in cholesterol that is far from Aβ (Zhao et al., 2011). The orientation of the lipid tails is also altered (Chang et al., 2010; Tofoleanu and Buchete, 2012)—they significantly point away from the region during protofibril interaction (Tofoleanu and Buchete, 2012). This equates to the area per lipid and cholesterol increasing (Qiu et al., 2011). In human brains afflicted with AD compared to normal brains no alteration in the content of lipid rafts are observed (Martín et al., 2010). Even though the composition is not altered, the changes on the molecular level have a more important effect on the cellular membrane.

One of the effects observed is the change in the cell membrane's fluidity, which inflates due to Aβ interaction (Hou et al., 2008; Khalifat et al., 2012; Liguori et al., 2013; Sasahara et al., 2013). In studies involving mitochondrial membrane vesicle model and rats, this was especially observed. The inner membrane of the mitochondria, which is the location of the final stage of ATP production, has cristae-like folds. These folds could not form *in vitro* under the influence of Aβ and may be due to the decrease in membrane fluidity and changes in dynamic friction of the cellular membrane (Khalifat et al., 2012). *In vivo* studies show similar findings, which reduced the function of cytochrome C oxidase (Hou et al., 2008). Finally, analysis of lipid raft microdomains in human AD brains elucidated that lipid rafts have a reduced unsaturated index and an increase in saturated fatty acids, resulting in more of the liquid-ordered phase and an increase in the cell membrane's viscosity (Martín et al., 2010). This parallels the stiffening, or Young's modulus, of the lipid bilayer, which has been shown to occur due to Aβ_1−42_ oligomer (Lulevich et al., 2010).

For neuronal cells the ability for the cell to be excited is important in relation to cognitive function. Aβ_1−42_ oligomer can increase the bilayer membrane conductance in both model and cell membranes (Sokolov et al., 2006; Lioudyno et al., 2012), specifically shown through the kinetic properties of one voltage-gated ion channel, the Kv 1.3 channel, being affected (Lioudyno et al., 2012). Besides directly affecting ion channels, the membrane itself is altered in Purkinje cells by soluble Aβ_1−42_ and thus reduces the intrinsic membrane excitability (Hoxha et al., 2012). Both Aβ_1−40_ (Sabaté et al., 2012) and Aβ_1−42_ (Choucair et al., 2007) interacting with liposomes cause their membrane to become leakier. Only the soluble but not the fibrillar form of Aβ can cause a change in the membrane conductance Sokolov et al., 2006; Sabaté et al., 2012. It has been proposed that this is due to a combination of the thinning, Aβ-induced local defects, and an increase in the dielectric constant of the cellular membrane, which varied due to the composition of the lipid and not by cholesterol. Negatively charged lipid reduces the effects that Aβ has on the membrane conductance, while neutral membrane has no influence. This was proposed to be due to electrostatic repulsion since Aβ has a net charge of −3 (Sokolov et al., 2006). A theory has also been established stating that Aβ can make a pore in certain conditions upon insertion into the cellular membrane. Pore formation has been shown to be thermodynamically feasible with MD simulations, in which a beta-barrel can take form in the bilayer to potentially increase the leakiness (Sabaté et al., 2012). This theory is not widely accepted though, but is still under consideration for potential mechanisms of action to elucidate the observation of the conductance and excitability changes.

The repercussions of Aβ morph the healthy state of the cell in ways not limited to this review. The alterations to the membrane's biophysics upset the cell's normal function, which in turn evoke additional complications. While many of these issues induced by Aβ have been elucidated, a quandary still exists over the full understanding of membrane interactions of Aβ in AD.

On the other hand, physical properties of cellular membranes are most likely altered in AD not only due to direct effects caused by Aβ, but also via downstream cellular signaling involving oxidative stress and Aβ-induced oxidant pathways. Reactive oxygen species (ROS) are generated in response to various physiologic or pathologic conditions, and is one of the pathological factors in Alzheimer's disease (AD). In addition to ROS produced extrinsically, a cell may produce ROS as a result of normal metabolism and signaling processes. When excess ROS are present within the cell, this oxidative stress may have profound deleterious effects on the cell, including the direct oxidation of biomolecules (e.g., lipid, protein, and DNA), indirect alteration in cellular structures and functions, and the induction of cell death. Direct interaction between ROS and the cell membrane can lead to the alteration of membrane biophysical properties, such as membrane molecular order and membrane fluidity. Our previous data demonstrated H_2_O_2_ made membranes more molecularly-ordered (or gel-like) in astrocytes and was a combined effect of direct oxidation and indirect alterations mediated by MAPK pathway (Zhu et al., 2005). Additional evidence showed that oxidant menadione also changed plasma membrane molecular order and fluidity, making it more gel-like (Zhu et al., 2006). Both NADPH oxidase and phospholipase A_2_ (PLA_2_) regulated such alterations in addition to direct oxidation from ROS.

Thus, cellular membrane's biophysics, defined here as the molecular order, fluidity or viscosity, and the surface charge, play a critical role in the manipulation of Aβ 's confirmation, aggregation, and their direct interaction with the cell membrane. In turn, the residues of Aβ, which are Aβ_25−36_, Aβ_1−40_, Aβ_1−42_, as well as reactive oxygen species, also affect the CM's biophysical properties (Table 2). These events are speculated to be one of the keys factors in the initiation of the AD cascade, therefore becoming a target for potential therapeutic strategies (Mckoy et al., 2012; Lee et al., 2014).

**Table 2:** **The influence of Aβ on cell membrane's composition and biophysics**.

**Effects of Aβ on cell membrane**	**References**
Introduce voids, making membrane more molecularly disordered	Chang et al., 2010
Increase H_2_O in membrane, leading to thinner lipid bilayer	Hicks et al., 2008
Decrease in membrane fluidity	Hou et al., 2008; Khalifat et al., 2012; Liguori et al., 2013; Sasahara et al., 2013
Increase in membrane viscosity	Martín et al., 2010
Increase in membrane stiffness	Lulevich et al., 2010
Increase in bilayer membrane conductance	Sokolov et al., 2006; Lioudyno et al., 2012
Introduce leakage in membrane	Choucair et al., 2007
ROS induced by Aβ make membrane more gel-like	Zhu et al., 2005, 2006

## Membrane biophysics and composition on APP processing

As discussed in the previous section, the physical properties of cellular membranes can be altered by Aβ interactions and Aβ-induced cellular pathways. In turn, these membrane alterations can have an impact on cellular functions, such as amyloid precursor protein (APP) processing and cerebral endothelial adhesion and permeability.

Two competing processing pathways are currently known for APP. In the amyloidogenic pathway, Aβ is derived from a proteolytic process of amyloid precursor protein (APP), in which APP is cleaved sequentially by β- and γ-secretases (Haass et al., 2012). Alternatively, APP can be cleaved by α-secretases between amino acids 16 and 17 within the Aβ domain to produce neurotrophic and neuroprotective soluble APP (sAPP_α_) in the non-amyloidogenic pathway (Thornton et al., 2006). The enhancement of APP processing by α-secretases has been suggested as a potential therapeutic strategy for AD (Cheng et al., 2007). Since APP, α-, β-, and γ-secretases are membrane proteins, APP processing is affected by the local membrane environment. In this section, we review the evidence about the effects of fatty acids and cholesterol on membrane properties and APP processing. Understanding the mechanisms leading to changes of membranes biophysics and how they result in changes in APP processing has the potential to provide insights into new therapeutic strategies for prevention and treatment of AD.

Fatty acids are important ingredients in various dietary sources and play a central role in the normal development and function of the brain (Dyall and Michael-Titus, 2008; Schuchardt et al., 2010). Long-chain ω-6 and ω-3 polyunsaturated fatty acids (PUFAs), the major polyunsaturated fatty acids in the central nervous system, are essential for prenatal brain development and normal brain functions (Uauy et al., 2001; Dyall and Michael-Titus, 2008). The disturbed metabolism of fatty acids is associated with AD. For instance, lower levels of docosahexaenoic acid (DHA) have been reported in serum samples taken from AD patients (Tully et al., 2003), while greater consumption of DHA has significantly reduced the likelihood of developing AD (Schaefer et al., 2006). In 15-month-old APP/PS1 mice, DHA supplementation improved spatial memory, decreases Aβ deposition, and slightly increased relative cerebral blood volume, indicating that a DHA-enriched diet could diminish AD-like pathology (Hooijmans et al., 2007). On the other hand, accumulation of trans fatty acids in the cellular membrane increased production and oligomerization of Aβ (Grimm et al., 2011).

PUFAs in neuronal cells can influence cellular functions through effects on membrane properties (Hibbeln et al., 2000; Sinclair et al., 2007; Heinrichs, 2010). The ability of fatty acids to modulate membrane properties and functions depends on both the saturation degree of the fatty acids and the trans/cis ratio of the unsaturated fatty acids (Loffhagen et al., 2004; Yang et al., 2011). For example, diets enriched in unsaturated PUFAs, DHA, and AA, have been shown to increase membrane fluidity of neurons and other cells (Mclauren Dorrance et al., 2000; Horrocks and Farooqui, 2004; Hashimoto et al., 2006; Fukaya et al., 2007). DHA was capable of counteracting cholesterol-induced decrease in platelet membrane fluidity and modulating platelet hyperaggregation (Hashimoto et al., 2006). Similarly, cis-polyunsaturated linolenic, α-linoleic, and eicosatrienoic fatty acids increased membrane fluidity (Kitagawa et al., 1990). In contrast, incorporation of saturated fatty acids into membrane led to decreased membrane fluidity (Calder et al., 1994).

It has been reported that an increase in membrane fluidity leads to an increase in non-amyloidogenic cleavage by α-secretase to produce sAPPα (Kojro et al., 2001; Peters et al., 2009). Consistently, enrichment of cell membranes with PUFAs increases membrane fluidity and, subsequently, promotes non-amyloidogenic processing of APP (Yang et al., 2011). A typical Western diet (with 40% saturated fatty acids and 1% of cholesterol) fed to transgenic APP/PS1 mice increased Aβ, while diets supplemented with DHA decreased Aβ levels (Lim et al., 2005; Oksman et al., 2006). Similarly, DHA decreased the amount of vascular Aβ deposition (Hooijmans et al., 2007) and reduced cortical Aβ burden in the aged Alzheimer mouse model. In this model, DHA modulated APP processing by decreasing both α- and β-APP C-terminal fragment products and full-length APP. DHA stimulated non-amyloidogenic APP processing resulting in reduced Aβ levels in cellular models of Alzheimer's disease (Sahlin et al., 2007). DHA can decrease cholesterol *de novo* synthesis, shift its distribution from raft to non-raft domains, and decrease β- and γ-secretase activity (Grimm et al., 2011). Meanwhile, our study of the effects of fatty acids on cell membrane fluidity and sAPPα secretion in relation to degrees of unsaturation has suggested that not all unsaturated fatty acids, but only those with 4 or more double bonds, such as arachidonic acid (20:4), eicosapentaenoic acid (20:5), and DHA (22:6), increased membrane fluidity and led to an increase in sAPPα secretion (Yang et al., 2011). Moreover, another study indicated that treatment of PSwt-1 CHO cells with oleic acid and linoleic acid increased γ-secretase activity and Aβ production (Liu et al., 2004). These studies suggest that modulation of PUFAs content in cellular membrane is essential in regulating sAPPα production partially due to their effects on membrane fluidity.

As previously mentioned, cholesterol is another essential component of the cellular membrane, which is mostly condensed in lipid rafts. Membrane cholesterol levels can be modulated by specific inhibitors of cellular biosynthesis such as statins, or it can be selectively extracted from plasma membrane by methyl-β-cyclodextrin (MβCD). The content of cholesterol in phospholipid bilayers affects many biophysical parameters of lipid bilayers, such as thickness, thermo-mechanical properties, molecular packing, conformational freedom of phospholipid acyl chains and water, molecular oxygen permeability, membrane hydrophobicity, membrane excitability in neurons, internal dipolar potential, and membrane fluidity (Chen et al., 1999; Dumas et al., 1999; Socaciu et al., 2000; Hao et al., 2004; Arrais and Martins, 2007; Halling et al., 2008; Fantini and Yahi, 2010; Wang and Schreurs, 2010).

Intracellular cholesterol homeostasis regulates APP processing (Burns and Rebeck, 2010). A model of membrane compartmentalization has been suggested for APP in two cellular pools, one associated with the cholesterol-enriched lipid rafts, where Aβ is generated, and the other outside of rafts (i.e., non-raft domains), where α-cleavage occurs (Ehehalt et al., 2003; Colell et al., 2009). It was reported that membrane cholesterol depletion decreased the content of APP in cholesterol and sphingolipid-enriched membrane microdomains and subsequently inhibited the amyloidogenic pathway to produce Aβ (Kojro et al., 2001; Eckert et al., 2003). In contrast, cholesterol accumulation in the Niemann Pick type C (NPC) model cells has been shown to shift APP localization to lipid rafts (Kosicek et al., 2010). Consistent with the membrane compartmentalization model, cellular cholesterol depletion resulted in increased membrane fluidity (Kojro et al., 2001; Colell et al., 2003; Luneva et al., 2007; Rog et al., 2008). In turn, increase in membrane fluidity shifts APP processing to non-amyloidogenic cleavage by α-secretase (Galbete et al., 2000; Colell et al., 2003; Abad-Rodriguez et al., 2004; Xiu et al., 2006; Luneva et al., 2007; Kosicek et al., 2010). The removal of cholesterol with Mβ CD or treatment with lovastatin increased membrane fluidity, which resulted in higher expression of the α-secretase and impaired internalization of APP (Kojro et al., 2001). The increased membrane fluidity also correlated with redistribution of cholesterol, sphingomyelin, and proteins involved in APP processing between raft and non-raft domains, and enhanced sAPPα production (Clement et al., 2010). At the same time, cholesterol enrichment has been shown to reduce membrane fluidity (Hashimoto et al., 2006; Buffone et al., 2009). Exposure of cholesterol to astrocytes, primary neurons, and glial cultures inhibited the secretion of sAPPα and reduced cell viability (Racchi et al., 1997; Galbete et al., 2000; Xiu et al., 2006). Furthermore, some studies showed that cholesterol levels in the membranes were positively correlated with β-secretase activity (Liu et al., 2009), while lovastatin enhanced α-secretase activity (Xiu et al., 2006). Cholesterol enrichment that impeded membrane fluidity may lower sAPPα production by hindering the interaction of the substrate with its proteases (Bodovitz and Klein, 1996). Interestingly, substitution of cholesterol by the steroid 4-cholesten-3-one induced minor change in membrane fluidity and reduced sAPPα secretion, whereas substitution of cholesterol by lanosterol increased membrane fluidity and sAPPα secretion (Kojro et al., 2001). These results suggest reversible effects of cholesterol on the α-secretase activity depending on membrane fluidity.

Many studies support the notion that Aβ production occurs in endosomes (Kinoshita et al., 2003; Small and Gandy, 2006; Cirrito et al., 2008; Rajendran et al., 2008; Schobel et al., 2008). APP internalization from the plasma membrane enhances APP cleavage by β-secretase to increase Aβ levels (Grbovic et al., 2003). In contrast, APP, lacking its cytoplasmic internalization motif, accumulates at the plasma membrane and undergoes cleavage by α-secretase (Haass et al., 1993; Koo and Squazzo, 1994). Cholesterol increased clathrin-dependent APP endocytosis in a dose-dependent and linear manner (Cossec et al., 2010). Moreover, alterations in cholesterol transport from late endocytotic organelles to the endoplasmic reticulum had important consequences for both APP processing and the localization of γ-secretase-associated presenilins (Runz et al., 2002). An increased cholesterol level in AD has been suggested to be responsible for the enhanced internalization of clathrin-dependent endocytosis of APP and the overproduction of Aβ (Cossec et al., 2010). Alternatively, APP internalization could be reduced by lowering cholesterol, which led to an increase in membrane fluidity, APP accumulation on the cell surface, and increased sAPPα secretion (Kojro et al., 2001).

Thus, summarizing findings, discussed in this chapter we can conclude that increased membrane fluidity favors non-amyloidogenic processing of APP. In turn, membrane fluidity can be increased by unsaturated PUFA with for or more double bonds (DHE, arachidonic, and eicosapentaenoic acid), or by reduction of membrane cholesterol levels (Table 3).

**Table 3:** **Summary of the different treatments on membrane fluidity, accumulation of APP at cell surface, and secretion of sAPPα and Aβ**.

**Treatment**	**Membrane fluidity**	**APP at cell surface**	**Secretion of sAPP α**	**Aβ**	**References**
DHA	↑	NA	↑	↓	Lim et al., 2005; Hooijmans et al., 2007; Sahlin et al., 2007; Yang et al., 2011
EPA	↑	NA	↑	NC	Yang et al., 2011
AA	↑	↑	↑	NC	Yang et al., 2010, 2011
MβCD	↑	↑	↑	↓	Kojro et al., 2001
Cholesterol	↓	↓	↓	↑	Galbete et al., 2000; Colell et al., 2003; Xiu et al., 2006; Luneva et al., 2007; Rog et al., 2008; Buffone et al., 2009; Cossec et al., 2010; Marquer et al., 2011
C_6_H_5_OH	↑	NA	↑	↓	Peters et al., 2009
PF68	↓	NA	↓	↑	Peters et al., 2009
Aβ	↓	NA	NC	↑	Peters et al., 2009

*↑ denotes increase, ↓ denotes decrease, NC denotes no change, NA denotes data not available. Abbreviations: DHA, docosahexaenoic acid (22:6); EPA, eicosapentaenoic acid (20:5); arachidonic acid, AA (20:4); ALA, α-linolenic acid (18:3); MβCD, methyl-β-cyclodextrin; C_6_H_5_OH, benzyl alcohol; PF68, pluronic F68*.

## Membrane adhesion properties and permeability of the blood brain barrier in AD

Significant body of evidence indicates that BBB dysfunction plays critical role in the development and progression of AD (Snyder et al., 2014; Sweeney et al., 2015). In the early stage of AD, microvasculature deficiencies, inflammatory reactions, Aβ surrounding the cerebral vasculature and endothelial dysfunctions are commonly observed (Borroni et al., 2002; Montagne et al., 2015). Continuous neurovascular degeneration and accumulation of Aβ on blood vessels resulting in cerebral amyloid angiopathy is associated with further progression of the disease and cognitive decline. One of the features of AD brains is an accumulation of monocytes in the vessel walls and of activated microglia cells in the adjacent parenchyma, which has been found to correlate with increased deposition of Aβ in the cerebral vasculature (Maat-Schieman et al., 1997; Uchihara et al., 1997). It has been shown that peripheral monocytes can migrate across the blood brain barrier (BBB) and differentiate into microglia within the brain parenchyma (Mezey et al., 2000). *In vitro* studies have demonstrated that Aβ deposition at the endothelial cell layer enhances the transmigration of monocytes (Francisco and Gonzalez-VelasquezMoss, 2008). Thus, increased transmigration of monocytes past the BBB is thought to drive the disease development toward exacerbation of the oxidative and inflammatory conditions characteristic of the AD brain.

**Figure 2:**
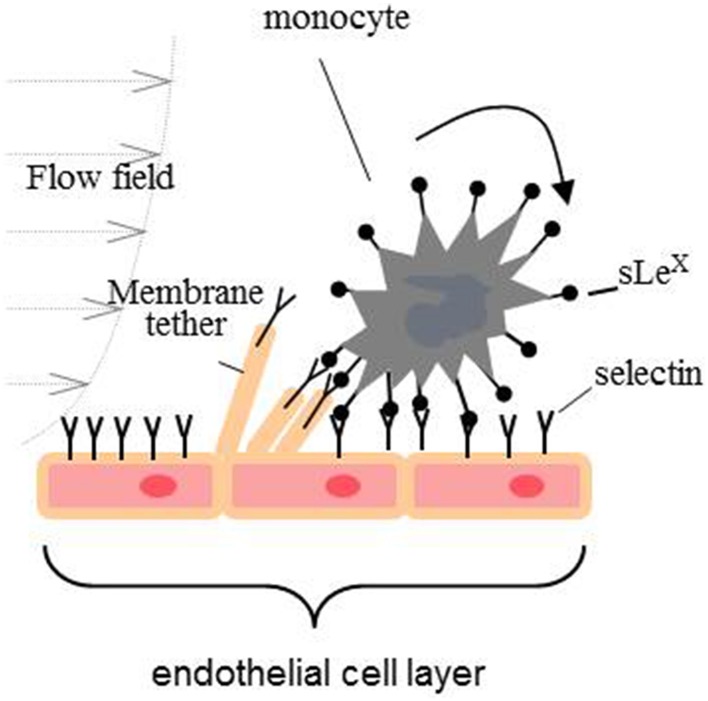
**Primary capture of monocytes to the endothelium and rolling**.

Transmigration of monocytes is a sequential process having at least three distinct adhesive events: capture, tethering, and rolling; firm adhesion and arrest; and crawling on the endothelial surface to find an intercellular junction for transmigration to the target tissue. Primary capture to the endothelium and rolling is a very dynamic process mediated cooperatively by the adhesive bond between adhesion molecules (mainly P-, E-, and L-selectins) and their ligand, the shear stress imposed by blood flow, and the mechanical properties of the endothelial membrane (Figure 2) (Alon et al., 1997; Marshall et al., 2003; Girdhar and Shao, 2004; Sun et al., 2007). The adhesion energy between selectins and their ligands is characterized by fast binding on and off rates. If the adhesive bond pulling force is greater than 25 pN, the off rate of selectin-ligand bond will be increased (slip-bond). In contrast, if a pulling force is smaller than 25 pN, the off rate will be decreased (catch-bond) (Marshall et al., 2003). During the rolling process, the pulling force imposed on the adhesive bond by the blood flow acts upon on the monocyte and endothelial cell membrane. If a pulling force greater than 50 pN is imposed on an endothelial surface, a tether (a cylindrical membrane tube that is tens of nanometers in diameter) will be extracted from the plasma membrane surface (Figure 2) (Girdhar and Shao, 2004). Earlier research has demonstrated that a lower membrane tether extraction force favors rolling (Girdhar and Shao, 2004; Girdhar et al., 2007). In this regards, it has been shown that enrichment of endothelial cells with cholesterol or treatment with Aβ_1−42_ oligomers reduces the adhesion force for CECs, increases the expression of cell adhesion molecules and probability of adhesion leading to enhanced rate of the monocytes' transmigration (Sun et al., 2007; Askarova et al., 2013).

The biomechanical characteristics of the tight junctions is another important factor, which maintains brain homeostasis and impermeability of BBB (Vedula et al., 2005, 2009). Tight junctions are responsible for the separation of an apical and basolateral domains of cell membrane and cell polarization. The molecular biology of tight junctions has been found to be very complex, and, among all, the structure of the tight junctions in cerebral endothelium is the most elaborate. Generally, molecular structure of tight junctions is presented by several classes of transmembrane and submembrane proteins. The transmembrane proteins are occludin, claudins, junction-adhesion molecules (JAM), endothelial cell-selective molecule (ESAM), and coxsackie- and adenovirus receptor (CAR). Submembrane proteins are presented by adaptor proteins first or second order. First order adaptors are directly associated with transmembrane proteins and include ZO-1, ZO-2, and ZO-3. Cingulin and coiled-coil protein are the second order adaptors which are characterized by indirect connection with transmembrane tight junction proteins (Wolburg et al., 2009).

In fact, the structure and biophysical characteristics of the tight junctions are strongly affected in the cerebrovascular cells of AD patients (Bednarczyk and Lukasiuk, 2011). In an animal model of AD, a cholesterol-enriched diet down-regulated the expression of the occluding and ZO-1, which was strongly correlated with an elevated level of BBB leakage (Chen et al., 2008). *In vitro*, treatment of primary rat CECs with Aβ_1−42_ for 3 days altered the expression of occluding and claudin-1, causing the relocation of plasma membrane subunits of claudin-5 and ZO-2 to the cytoplasm. At the same time, the cytoplasmic ZO-1 and ZO-2 where evenly distributed along the plasma membrane to the points of the cell-cell contacts (Marco and Skaper, 2006).

Apolipoprotein E (apoE), a major apolipoprotein in the brain, has been shown to be involved in tight junction alteration as well (Nishitsuji et al., 2011). ApoE is a polymorphic glycoprotein playing an important role in the transportation of lipids and lipid acceptors. ApoE exists in three isoforms – ApoE2, ApoE3, and ApoE4, and among these three isoforms, ApoE4, is the greatest risk factor for late-onset AD and Aβ-induced neuroinflammation (Halliday et al., 2015; Tai et al., 2015). *In vitro* study has demonstrated that the barrier functions of tight junctions were impaired when the CECs were reconstituted with primary astrocytes from apoE4-knock-in mice. In particular, the phosphorylation of occludin and the activation of protein kinase C (PKC)η in CECs were attenuated (Nishitsuji et al., 2011).

In turn, cell membrane biophysical properties are highly dependent on the F-actin network condition and the interacting membrane and cytoskeleton integrity (Khatibzadeh et al., 2013). Aβ has been shown to cause formation of actin stress fibers, induce actin polymerization and increase overall cell stiffness, but significantly soften the cells in the vicinity of the plasma membrane (Mendoza-Naranjo et al., 2007; Askarova et al., 2013). To support the data that Aβ oligomers affect tight junction, at least partially, via altered integrity of actin network within CECs, it has been demonstrated that the force needed for separation of cellular adhesion formed by tight junction proteins significantly decreases after treating cells both with Aβ and Cytochalasin-D (the actin disrupting agent) (Vedula et al., 2009). These findings suggest that the effects of Aβ on actin and tight junction proteins expression and spatial distribution, cause the alteration of tight junctions' biophysics and contribute to the BBB leakage.

Strong evidence exists that cerebral endothelium regulates clearance of internal Aβ across the BBB and influx of external Aβ into brain (Deane et al., 2009). Aβ influx into brain from the apical surface of CEC is regulated by receptor for advanced glycosylated end products (RAGE), while lipoprotein receptor related protein (LPR1) at the basolateral surface is responsible for its efflux. In AD brains there is significant imbalance between these two processes leading to the elevated level of Aβ in the brain's interstitial fluid and parenchyma (Deane et al., 2004, 2009). It has also been shown that CECs internalize Aβ differently compared to other cells of the central nervous system (Kandimalla et al., 2009). For example, neurons internalize Aβ primarily via non-endocytotic and energy independent pathway, while CECs demonstrate energy dependent endocytotic uptake (Kandimalla et al., 2009). Additionally, kinetics of endocytosis strongly depends on the biophysical properties of the plasma membrane: increasing membrane microviscosity slows down and eventually blocks membrane endocytosis of Aβ in different cell models (Elguindi et al., 1985; Callaghan et al., 1990). The data suggest that manipulations of the Aβ binding sites one the membrane and regulation of the CECs' plasma membrane biophysical properties may result in decreased Aβ internalization and endocytosis (Callaghan et al., 1990).

Thus, investigations of the effects of Aβ, cholesterol and ApoE isoforms on alterations of CECs' membrane biophysics, including imbalance in cell-cell adhesion, tight junctions integrity, CECs' endocytosis, and other important aspects of cell functions, would provide insights into the mechanisms of neuroinflammation in AD, their correlations with cardiovascular disorders, and may offer new therapeutic strategies for AD patients.

## Conclusions

Alterations of physical properties and lipid composition of cellular membranes have been found to impact cellular pathways and processes in many pathologic events of AD. In particular, membrane's biophysical properties play a critical role in the manipulation of Aβ 's confirmation, aggregation, and their direct interaction with the cell membrane. In turn, Aβ species, as well as reactive oxygen species, also affect the CM's biophysical properties in different ways. Moreover, these membrane alterations have an impact on other cellular functions, such as amyloid precursor protein processing and cerebral endothelial adhesion and permeability. Yet the involvements of membrane biophysics in many cellular processes, e.g., Aβ influx and efflux across the BBB, and NADPH oxidase activations in AD, are still understudied. The research direction focusing on the involvements of cellular membranes in AD should provide insightful information for the development of treatment strategies against AD.

### Conflict of interest statement

The authors declare that the research was conducted in the absence of any commercial or financial relationships that could be construed as a potential conflict of interest.
